# Association between hidradenitis suppurativa and atopic diseases: a multi-center, propensity-score-matched cohort study

**DOI:** 10.7150/ijms.90086

**Published:** 2024-01-01

**Authors:** Hui-Chin Chang, Chen-Yu Lin, Yu-Chen Guo, Hsin-Yo Lu, Chien-Ying Lee, Meng-Che Wu, Shuo-Yan Gau

**Affiliations:** 1 Evidence-based Medicine Center, Chung Shan Medical University Hospital, Taichung, Taiwan.; 2 Library, Chung Shan Medical University Hospital, Taichung, Taiwan.; 3 School of Medicine, Chung Shan Medical University, Taichung, Taiwan; 4 Department of Pharmacy, Chung Shan Medical University Hospital, Taichung, Taiwan.; 5 Department of Pharmacology, Chung Shan Medical University, Taichung, Taiwan.; 6 Division of Gastroenterology, Children's Medical Center, Taichung Veterans General Hospital, Taichung, Taiwan.; 7 Department of Post-Baccalaureate Medicine, College of Medicine, National Chung Hsing University, Taichung, Taiwan.; 8 Pediatric Inflammatory Bowel Disease Center, Massachusetts General Hospital for Children, Boston, MA, USA; 9 Institute of Medical Education, Chi Mei Medical Center, Tainan, Taiwan

**Keywords:** hidradenitis suppurativa, atopic dermatitis, cohort, epidemiology, electronic medical records

## Abstract

**Background:** Cross-sectional evidence has suggested a high prevalence of atopic diseases in patients with hidradenitis suppurativa (HS). However, there is a lack of evidence based on longitudinal studies. This study aimed to assess the risk of different atopic diseases, including asthma, atopic dermatitis, and allergic rhinitis, in patients with HS.

**Methods:** In this retrospective cohort study, data from the TriNetX research network were obtained. Patients with HS were enrolled, and a 1:1 propensity score matching was performed to select a non-HS control group. Matching covariates included age, sex, race, comorbidities, comedications, socioeconomic status, lab data, and medical utilization status. Hazard ratios (HR) for atopic diseases were assessed.

**Results:** Over a 15-year follow-up period, patients with HS were found to be at a higher risk for atopic dermatitis (HR = 1.65; 95% CI, 1.44-1.90), asthma (HR = 1.41; 95% CI, 1.33-1.49), and allergic rhinitis (HR = 1.08; 95% CI, 1.03-1.13). A similar trend was observed in shorter follow-up periods. The association between HS, atopic dermatitis, and asthma was consistent across different age and sex subgroups.

**Conclusion:** Atopic diseases including atopic dermatitis, asthma and allergic rhinitis are associated with HS. Further investigation is needed to assess the necessity of early screening for atopic diseases in patients with HS.

## Introduction

As a chronic inflammatory skin condition, hidradenitis suppurativa (HS) typically affects areas rich in sweat glands, including the groin, breasts, and perineum 1. Despite its relatively low prevalence, ranging from 0.00033% to 4.10%, HS substantially affects people's quality of life due to its chronicity and potential systemic implications 2. In particular, HS exhibits gender and racial disparities, disproportionately affecting young women, particularly those of African-American descent 3.

Concomitantly, atopic diseases, including allergic rhinitis, allergic dermatitis, and asthma, globally pose a health burden for 30-40% of the worldwide population 4. These conditions manifest themselves through a dominant immune response of type 2 T (Th2), orchestrated by cytokines such as IL-4 and IL-13^5^. Although HS has historically been associated with distinct inflammatory pathways characterized by cytokines like IL-1β, TNFα, IFN-γ, and IL-17/22, a recent meta-analysis provided evidence regarding the association between HS and atopic dermatitis and asthma, challenging the conventional separation of these conditions 1, 6.

A recent large-scale cohort study shed light on the intricate interplay between HS and atopic dermatitis, suggesting a bidirectional connection. It indicated that individuals with HS had a twofold higher likelihood of developing atopic dermatitis 7. Moreover, another recent study proposed a causal link between the two conditions, implicating the potential role of HS in the development of atopic dermatitis 8. These revelations accentuate the complex nature of immune interactions underlying these disorders. In addition, a study highlighted a plausible correlation between heightened apoptosis of type 1 T-helper cells (TH1), responsible for producing elevated levels of IFN-γ, and the prevailing TH2 predominance characteristic of atopic diseases 9. This mechanistic insight introduces an additional dimension to the immune interplay, suggesting the existence of shared pathways between these entities.

To the best of our knowledge, the real-world association between HS and atopic diseases has not been fully explored. Furthermore, the available evidence is predominantly derived from cross-sectional studies, which do not offer insights into the risk of developing atopic diseases in HS patients within a longitudinal study design. To address this gap, we conducted a multi-center, propensity score-matched cohort study.

## Methods and Materials

We obtained data from the global TriNetX research network, which compiles electronic medical records from collaborative healthcare organizations (HCOs) worldwide. TriNetX has been previously utilized for investigating the relationships between exposures and outcomes in prior studies 10-12. Specifically, we employed the US collaborative network, a subset within the TriNetX research network, which sources its data from American healthcare organizations. This dataset includes records from 60 healthcare organizations across the United States. Our definitions for exposure, outcome, and covariates were established using administrative codes, such as ICD-10-CM codes for diseases, all of which are available within the TriNetX research network. Additional algorithm details are provided in the **supplementary file**. In the TriNetX research network, missing data for variables such as sex or race were categorized as 'unknown sex and 'unknown race' within the system. These missing data were not excluded from subsequent analyses, and imputation was not conducted. The information in the current dataset was properly de-identified in compliance with the regulations outlined in Section §164.514(a) of the HIPAA Privacy Rule. All processes and ethical considerations were reviewed by a qualified expert, as defined in Section §164.514(b)(1) of the HIPAA Privacy Rule, and this formal assessment negated the necessity for IRB permission or a waiver in studies conducted using the TriNetX research network 13, 14. This study adhered to the STROBE guideline and the TriNetX publication guideline. All analyses were executed through the analytical system within the TriNetX research network on November 11th, 2023. No direct interaction or intervention with human participants was undertaken in this study.

All participants were enrolled during the study period, spanning from January 1, 2005, to December 31, 2017. Exclusions were applied to individuals under 18 years old, those who had passed away before the index date, and those with a history of cancer or atopic diseases (including asthma, atopic dermatitis, allergic rhinitis, and conjunctivitis) prior to the index date. The HS group consisted of individuals diagnosed with HS before the index date, while the control group comprised those who underwent general examinations and had no prior HS diagnosis. Propensity score matching was performed in a 1:1 ratio, aligning critical covariates such as age, sex, race, comorbidities, comedications, socioeconomic status, lab data, and medical utilization status. The study's outcome event was defined as incident atopic diseases, excluding events occurring between the index date and 6 months thereafter. Following 1:1 PSM, the study included 40,053 HS patients and 40,053 controls for assessment. Hazard ratios (HR) for various atopic diseases in the HS group compared to the controls were assessed. Stratification analyses were conducted based on age and sex subgroups to further examine the observed associations. Different matching algorithms and wash-out periods were applied as sensitivity analyses to mitigate potential biases.

All statistical analyses were conducted using the TriNetX research network system. For each analysis, we calculated a 95% confidence interval (95% CI) to indicate the significance of the HR value. To assess the baseline characteristics of participants and the significance between the control group and HS group, we used the standardized difference (SD). When the presented SD value exceeded 0.1 between the groups, the difference was deemed statistically significant.

## Results

The study included a population of 40,053 HS patients and an equal number of matched control individuals. The mean age of the HS patients was 34.2 years. Within the HS group, 27.0% were male, and 72.3% were female. The HS group predominantly consisted of white and Black or African American individuals, accounting for 45.4% and 35.0% of the group, respectively. Before matching, there was a significant difference in the baseline distribution of diabetes mellitus between the HS and control cohorts. However, most other comorbidities, including hypertension and hyperlipidemia, did not show significant differences before matching. Following matching, the baseline disparities between the HS and non-HS groups became insignificant (**Table 1**).

### Risk of atopic dermatitis, asthma and allergic rhinitis

Compared to non-HS controls, HS patients were associated with a higher risk of developing atopic dermatitis. In the short-term follow-up (3 years), HS patients exhibited a 1.84-fold risk of atopic dermatitis (95% CI, 1.47-2.31), a 1.41-fold risk of asthma (95% CI, 1.33-1.49), and a 1.08-fold risk of allergic rhinitis (95% CI, 1.03-1.13). Over a 10-year follow-up, the HR for HS patients developing atopic dermatitis, asthma, and allergic rhinitis was 1.65 (95% CI, 1.43-1.90), 1.40 (95% CI, 1.32-1.49), and 1.08 (95% CI, 1.02-1.14), respectively. This risk persisted during the 15-year follow-up (**Table 1**). In the crude model without propensity score matching, the HR for atopic dermatitis was 1.87 (95% CI, 1.72-2.04), and the HR for asthma was 1.85 (95% CI, 1.78-1.92). This association also held in sensitivity models using various matching variables (**Table S1**). Additionally, the 15-year risk of atopic diseases in HS patients remained significant even when applying longer wash-out periods. When excluding incident atopic dermatitis cases diagnosed within 3 years after the index date, the 15-year risk of atopic dermatitis in HS patients was 1.57 times higher than in controls (95% CI, 1.34-1.85), and the risk of asthma was 1.39 times higher than in controls (95% CI, 1.30-1.48) (**Table S2**).

### Stratification analysis

The observed associations between HS and atopic dermatitis, as well as HS and asthma, were significant in both male and female HS subgroups. In male HS patients, the risk of developing atopic dermatitis and asthma in the subsequent 15 years was 1.56 (95% CI, 1.17-2.07) and 1.40 (95% CI, 1.22-1.60), respectively. For female HS patients, the HR for atopic dermatitis was 1.56 (95% CI, 1.34-1.82), and the HR for asthma was 1.42 (95% CI, 1.33-1.51). Similarly, in both the younger (18-64 years old) and older (above 65 years old) HS subgroups, the risk of developing asthma and atopic dermatitis was significantly higher compared to their respective control groups (**Table 3**).

## Discussion

Several studies have previously reported a close association between HS and atopic diseases. However, our study takes a step further by revealing a long-term association between HS, atopic dermatitis, asthma, and allergic rhinitis. Unlike previous investigations, which often relied on cross-sectional methods, our study offers robust long-term data, addressing the evidence gap in this field.

HS is known to be associated with various inflammatory comorbidities 15-17. Apart from inflammatory conditions, HS has also been linked to dysfunctions in different organ systems, including renal diseases, hypertension, dyslipidemia, and diabetes 16, 18. The pathogenesis of HS involves the IL-23, Th17 pathway, and Th1 pathway 19, 20. Excessive keratinization and epithelial hyperplasia of hair follicles lead to follicular obstruction 21. The presence of bacteria and Damage-Associated Molecular Pattern Molecules (DAMPs) in obstructed follicles triggers the activation of macrophages, resulting in the release of IL-1β and TNF 22. IL-1β facilitates the migration of neutrophils into the skin 23, while TNF increases the Th17/Treg ratio, leading to immune dysregulation and immune cell infiltration in HS patients 24. Dendritic cells produce IL-12 and IL-23, activating the Th1 and Th17 pathways, respectively 25, 26. This process eventually establishes the chronic inflammation cycle in HS.

Atopic dermatitis is influenced by both lifestyle and environmental factors, with adult prevalence reported to range from 7% to 14% worldwide 27-30. It primarily occurs between the ages of 20 and 40, with a higher incidence in females until the age of 65. The role of Th2 immunity in the immunological reactions of atopic dermatitis is well-established 31. Moreover, the etiology of atopic dermatitis varies among different ethnic groups 32, with Asian atopic dermatitis patients exhibiting activation of both Th2 and Th17 pathways, while those of Europeans and Africans primarily involve the Th2 pathway 33.

Asthma, a common clinical disease, has a prevalence ranging from 1% to 18%. In asthma patients older than 13 years, the prevalence is higher in females than in males 34. Apart from the typical Th2-mediated asthma, there are other forms of asthma induced by Th17 or Th1 pathways 35. It is evident that HS, atopic dermatitis, and asthma are all highly associated with immune dysregulation. They share a common mechanism involving the dysregulation of the Th17 pathway. Furthermore, recent research has suggested a potential genetic association between HS and atopic dermatitis 8, although further studies are needed to provide conclusive evidence.

Some data in our study warrants further consideration. Among HS patients, the risk of developing atopic dermatitis appeared to be numerically higher in those aged over 65 when compared to individuals aged 18 to 64. This could be attributed to the higher prevalence of atopic dermatitis in individuals over the age of 65 36. When we conducted stratification analysis by age, we excluded younger individuals, who tend to have a higher incidence of atopic dermatitis, potentially amplifying the association between older HS patients and atopic dermatitis.

Another noteworthy point concerns the elevated risk of atopic dermatitis in HS patients. While both atopic dermatitis and asthma are considered allergic diseases resulting from a Th1/Th2 imbalance, the risk of atopic dermatitis was numerically higher than that of asthma in HS patients. This observed trend aligns with findings in previous literature. In a previous meta-analysis, despite the high heterogeneity in the existing evidence, the odds ratio for developing atopic dermatitis in HS patients was calculated to be 4.10 (95% CI, 2.16-8.18), whereas the odds ratio for asthma was 1.50 (95% CI, 1.24-1.81) 6. In studies with a longitudinal design, a recent Korean study reported a 1.54-fold risk (95% CI, 1.47-1.61) for atopic dermatitis and a 1.21-fold risk (95% CI, 1.16-1.26) for asthma in HS patients 37. The higher risk of atopic dermatitis in HS may be partially attributed to a potential genetic link between the two conditions, as suggested by a two-sample Mendelian randomization study 8. However, current evidence remains insufficient to confirm the precise genetic association between HS and atopic dermatitis.

Our study has several notable limitations. Firstly, it is an observational study, and therefore, we cannot establish causation between HS, atopic dermatitis, and asthma. Secondly, despite our matching efforts, our study sample is predominantly composed of individuals of White and African ethnic backgrounds, with smaller proportions of Asian and Native American individuals. Given the variation in clinical disease presentation among different racial groups, the generalizability of our findings may be limited. Thirdly, due to data limitations, we were unable to classify HS patients by the severity of their condition, which hinders our ability to assess the impact of different disease states on our outcomes. Fourthly, despite our confounder matching and sensitivity analyses, misclassification bias and residual confounding may still exist, including the possibility of misdiagnosed HS or unaccounted potential confounders. Therefore, our results should be interpreted with caution. Notwithstanding these limitations, our findings suggest a potential long-term association between HS and atopic diseases, providing valuable evidence of HS as a risk factor for atopic diseases. This discovery may contribute to refining current HS treatment guidelines and increasing awareness of this clinical condition.

## Supplementary Material

Supplementary tables.

## Figures and Tables

**Figure 1:**
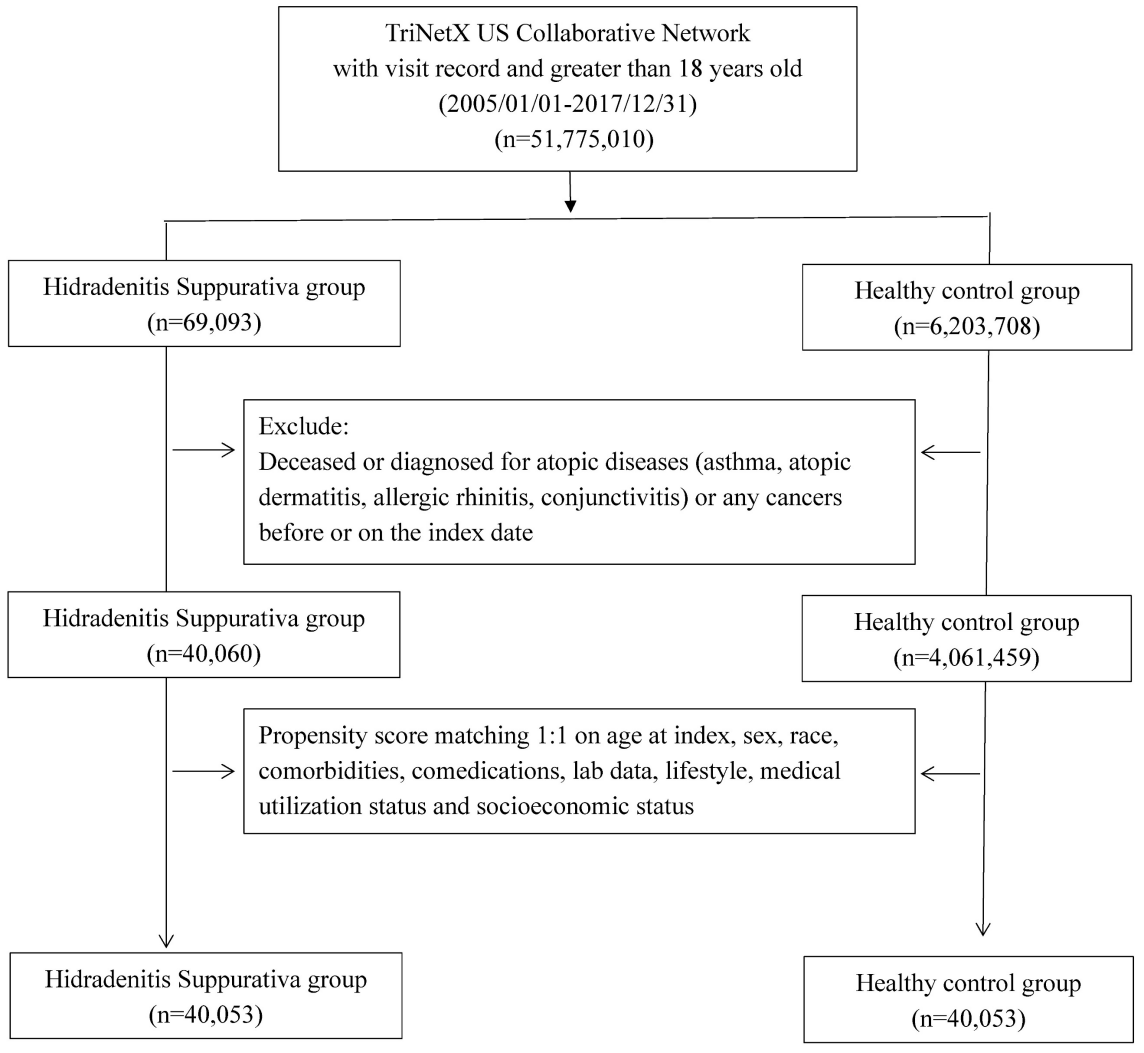
Flowchart of participant selection

**Table 1:** Baseline characteristics of study subjects (before and after propensity score matching)

	Before matching				After matching^a^		
	HS cohort (n=40,060)	Control cohort (n=4,061,459)	Std diff		HS cohort (n=40,053)	Control cohort (n=40,053)	Std diff
**Age at index**							
Mean±SD	34.2±14.2	39.3±20.4	**0.29**		34.2±14.2	34.6±14.6	0.02
**Sex, n (%)**							
Male	10798 (27.0)	1767975 (43.5)	**0.35**		10798 (27.0)	10753 (26.8)	0.00
Female	28976 (72.3)	2213467 (54.5)	**0.38**		28969 (72.3)	28980 (72.4)	0.00
Unknown sex	286 (0.7)	80017 (2.0)	**0.11**		286 (0.7)	320 (0.8)	0.01
**Race, n (%)**							
White	18186 (45.4)	2455819 (60.5)	**0.31**		18186 (45.4)	18123 (45.2)	0.00
Black or African American	13639 (34.0)	563360 (13.9)	**0.49**		13632 (34.0)	13889 (34.7)	0.01
Asian	615 (1.5)	136698 (3.4)	**0.12**		615 (1.5)	1073 (2.7)	0.08
American Indian or Alaska Native	182 (0.5)	12976 (0.3)	0.02		182 (0.5)	130 (0.3)	0.02
Native Hawaiian or Other Pacific Islander	54 (0.1)	5335 (0.1)	0.00		54 (0.1)	39 (0.1)	0.01
Other Race	1473 (3.7)	147230 (3.6)	0.00		1473 (3.7)	1291 (3.2)	0.02
Unknown Race	5911 (14.8)	740041 (18.2)	0.09		5911 (14.8)	5508 (13.8)	0.03
**Social economic status**							
Socioeconomic/psychosocial circumstances problem	526 (1.3)	21657 (0.5)	0.08		524 (1.3)	495 (1.2)	0.01
**Lifestyle**							
Alcohol dependence, smoking and substance use	3723 (9.3)	114918 (2.8)	**0.27**		3717 (9.3)	3751 (9.4)	0.00
**Comorbidities**							
Hypertension	4163 (10.4)	373134 (9.2)	0.04		4162 (10.4)	4200 (10.5)	0.00
Diabetes mellitus	2505 (6.3)	160770 (4.0)	**0.10**		2503 (6.2)	2467 (6.2)	0.00
Hyperlipidemia	2097 (5.2)	241296 (5.9)	0.03		2097 (5.2)	2012 (5.0)	0.01
Psoriasis	310 (0.8)	11973 (0.3)	0.07		308 (0.8)	304 (0.8)	0.00
Urticaria	297 (0.7)	13663 (0.3)	0.06		297 (0.7)	254 (0.6)	0.01
**Medications**							
Glucocorticoids	5656 (14.1)	310671 (7.6)	**0.21**		5650 (14.1)	5599 (14.0)	0.00
Adalimumab	149 (0.4)	2226 (0.1)	0.07		145 (0.4)	102 (0.3)	0.02
Infliximab	86 (0.2)	1392 (0.0)	0.05		84 (0.2)	60 (0.2)	0.01
**Medical Utilization Status**							
Emergency visit	8237 (20.6)	403967 (9.9)	**0.30**		8232 (20.6)	8342 (20.8)	0.01
Inpatient visit	5241 (13.1)	395464 (9.7)	**0.11**		5236 (13.1)	5270 (13.2)	0.00
**Laboratory data**							
BMI, n (%)							
≧ 35 (kg/m^2^)	2602 (6.5)	73254 (1.8)	**0.24**		2596 (6.5)	2622 (6.5)	0.00
C reactive protein, n (%)							
≧ 3 (mg/L)	1224 (3.1)	49620 (1.2)	**0.13**		1220 (3.0)	1122 (2.8)	0.01

Bold font represents a standardized difference was more than 0.1 HS: Hidradenitis Suppurativa; BMI, body mass index; SD, standardized difference ^a^Propensity score matching was performed on age at index, sex, race, status of comorbidities (including diabetes mellitus, chronic kidney disease, heart failure), status of smoking, alcoholism and substance use, medical utilization status, lab data regarding inflammation status (CRP) and social economic status (problems related to housing and economic circumstances, persons with potential health hazards related to socioeconomic and psychosocial circumstances).

**Table 2:** Risk of atopic diseases under different follow-up time^a^

Outcomes	Hazard ratio (95% Confidence interval)^b^		
	3 years	10 years	15 years
Atopic dermatitis	**1.84 (1.47,2.31)**	**1.65 (1.43,1.90)**	**1.65 (1.44,1.90)**
Asthma	**1.43 (1.31,1.56)**	**1.40 (1.32,1.49)**	**1.41 (1.33,1.49)**
Allergic rhinitis	**1.10 (1.02,1.19)**	**1.08 (1.02,1.14)**	**1.08 (1.03,1.13)**

HS: hidradenitis suppurativa ^a^Data present here were the value of follow up from 180 days after index date to the respective following up years. ^b^ Propensity score matching was performed on age at index, sex, race, body mass index, status of comorbidities (including diabetes mellitus, hypertension, hyperlipidemia, urticaria, psoriasis), status of comedication use (glucocorticoid, TNF alpha inhibitors), status of smoking, alcoholism and substance use, medical utilization status, lab data regarding inflammation status (CRP) and social economic status (problems related to housing and economic circumstances, persons with potential health hazards related to socioeconomic and psychosocial circumstances).

**Table 3:** Stratification analysis of age and sex

	New-onset atopic dermatitis			New-onset asthma			
Subgroups	HS cohort (No. of event/ HS patient amount in each subgroup)	Control cohort (No. of event/ non-HS patient amount in each subgroup)	HR (95% CI)^a^	HS cohort (No. of event/ HS patient amount in each subgroup)	Control cohort (No. of event/ non-HS patient amount in each subgroup)	HR (95% CI)^a^	
**Sex**							
Male	121/10794	77/10794	**1.56 (1.17,2.07)**	495/10794	358/10794	**1.40 (1.22,1.60)**	
Female	410/28971	262/28971	**1.56 (1.34,1.82)**	2340/28971	1667/28971	**1.42 (1.33,1.51)**	
**Age at index date**							
18-64 years old	485/36068	314/36068	**1.54 (1.33,1.77)**	2595/36068	1827/36068	**1.43 (1.35,1.52)**	
≥ 65 years old	55/3985	27/3985	**2.06 (1.30,3.27)**	278/3985	188/3985	**1.53 (1.27,1.84)**	

^a^ Propensity score matching was performed on age at index, sex, race, body mass index, status of comorbidities (including diabetes mellitus, hypertension, hyperlipidemia, urticaria, psoriasis), status of comedication use (glucocorticoid, TNF alpha inhibitors), status of smoking, alcoholism and substance use, medical utilization status, lab data regarding inflammation status (CRP) and social economic status (problems related to housing and economic circumstances, persons with potential health hazards related to socioeconomic and psychosocial circumstances).
