# Supplementary material for: Association between hidradenitis suppurativa and atopic diseases: a multi-center, propensity-score-matched cohort study
Source: Int J Med Sci. 2024 Jan 1;21(2):299–305. doi: 10.7150/ijms.90086 (PMC10758151; doi:10.7150/ijms.90086)
Supplement: Supplementary file 1 — Supplementary tables. [file ijmsv21p0299s1.pdf]

Supplementary Files

Utilized administrative codes in the current study

**Description**	**ICD-10-CM codes** [a]
Hidradenitis Suppurativa	L73.2
Neoplasms	C00-D49
Asthma	J45
Atopic dermatitis	L20
Allergic rhinitis	J30
Conjunctivitis	H10
Diabetes mellitus	E08-E13
Hypertension	I10
Hyperlipidemia	E78.5
Urticaria	L50
Psoriasis	L40
Socioeconomic and psychosocial circumstances	Z55-Z65
Substance abuse	F10-F19
Encounter for general examination	Z00
**Medications**	**ATC codes/ RxNorm**
Glucocorticoids	ATC code: H02AB
Adalimumab	RxNorm: 327361
Infliximab	RxNorm: 191831

[a]ICD-10-CM: International Classification of Diseases, Tenth Revision, Clinical Modification

1. **Supplementary Tables**

Table S1. Sensitivity analysis: risk of atopic diseases in HS patients based on different covariate matching models, with 15-year follow up

Outcomes	Hazard ratio (95% Confidence interval)			
	Crude	Model 1[a]	Model 2 [b]	Model 3 [c]
Atopic dermatitis	**1.87 (1.72,2.04)**	**1.86 (1.61,2.15)**	**1.80 (1.57,2.08)**	**1.78 (1.55,2.06)**
Asthma	**1.85 (1.78,1.92)**	**1.58 (1.49,1.68)**	**1.53 (1.44,1.62)**	**1.48 (1.40,1.57)**
Allergic rhinitis	**1.17 (1.13,1.21)**	**1.13 (1.07,1.19)**	**1.10 (1.05,1.16)**	**1.09 (1.03,1.14)**

[a] Propensity score matching was performed on age at index, sex, and race

[b] Propensity score matching was performed on age at index, sex, race and medical utilization status

[c] Propensity score matching was performed on age at index, sex, race and obesity status

Table S2. Sensitivity analysis: risk of atopic diseases with different wash-out periods [a]

Outcomes	Hazard ratio (95% Confidence interval)[b]		
	3 months	12 months	36 months
Atopic dermatitis	**1.67 (1.46,1.92)**	**1.65 (1.43,1.89)**	**1.57 (1.34,1.85)**
Asthma	**1.41 (1.33,1.49)**	**1.40 (1.32,1.48)**	**1.39 (1.30,1.48)**
Allergic rhinitis	**1.07 (1.02,1.13)**	**1.09 (1.04,1.15)**	**1.10 (1.04,1.17)**

[a] Incident events occurred within each wash-out period were excluded in the corresponding analysis

[b] Propensity score matching was performed on age at index, sex, race, body mass index, status of comorbidities (including diabetes mellitus, hypertension, hyperlipidemia, urticaria, psoriasis), status of comedication use (glucocorticoid, TNF alpha inhibitors), status of smoking, alcoholism and substance use, medical utilization status, lab data regarding inflammation status (CRP) and social economic status (problems related to housing and economic circumstances, persons with potential health hazards related to socioeconomic and psychosocial circumstances).
